# Mandibular Canines with Two Roots and Two Root Canals: Case Report and Literature Review

**DOI:** 10.1155/2017/8459840

**Published:** 2017-09-13

**Authors:** Hugo Plascencia, Álvaro Cruz, Gerardo Gascón, Beatriz Ramírez, Mariana Díaz

**Affiliations:** ^1^Endodontic Postgraduate Program, CUCS-CUAltos, University of Guadalajara, Guadalajara, JAL, Mexico; ^2^Research Institute in Biomedical Sciences, CUCS, University of Guadalajara, Guadalajara, JAL, Mexico; ^3^Endodontic Postgraduate Program, CUCS, University of Guadalajara, Guadalajara, JAL, Mexico

## Abstract

Usually, the mandibular canine only has one root and one root canal. However, there has been a noticeable increment in evidence showing variations in its morphology, such as the presence of two roots and two root canals. The aim of this article was to present a case of a mandibular canine with two roots and two root canals and to review the available literature on this anatomic variation. Root canal treatment of tooth #43 with such morphology was performed in a 47-year-old woman. Careful inspection of the preoperative radiograph indicated the presence of more than one canal. The 12-month follow-up showed normal periapical tissues, with no pain or tenderness. Literature review revealed that the overall prevalence of such root canal configuration is 5.7%, with a strong preference for female sex (87.5%). Although mandibular canines with two roots and two root canals are not common, clinicians should always anticipate the presence of possible variations. Therefore, timely diagnosis and meticulous exploration of such mandibular canines allow for planning of an individualized treatment protocol, tailored to their peculiar morphology, focused on avoiding excessive weakening or even perforation of the roots.

## 1. Introduction

The mandibular canine is a strategically important tooth in the dental arch. Its long and stable root is useful for prosthetic support due to its proprioceptive properties that regulate or guide masticatory function, combined with its role in occlusal guidance during the eccentric movements and posterior disocclusion [1]. Therefore, considerable effort is directed to its preservation, even though there may be diverse morphologic challenges.

Usually, the mandibular canine only has one root and one root canal [2]. However, since the beginning of the 21st century, due to introduction of new technologies related to intraoperative vision magnification and innovative radiological imaging systems, there has been a noticeable increment in the number of clinical studies,* in vitro/ex vivo* studies, and case reports that have revealed morphological variations, such as the presence of two roots and two root canals in the mandibular canine. Finding variations, such as this, is unpredictable and the clinician must assume that any mandibular canine could morphologize with variation, so that any unforeseen treatment complications related to unusual root canal anatomy can be avoided. The objective of this article was to present a case of a mandibular canine with two roots and two root canals and to perform a literature review regarding this anatomical variation.

## 2. Case Presentation

**Figure 1:**
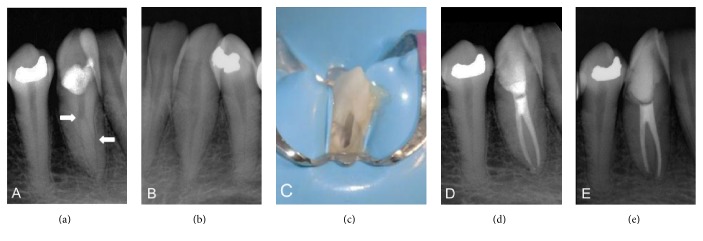
(a) Preoperative radiograph shows sudden loss in the continuity of the canal and the presence of a groove in the outer part of the root* (arrows)*. (b) Counterpart canine radiograph reveals typical root features. (c) Buccal access cavity due to dental giroversion. (d) Post-treatment radiograph. (e) 12-month follow-up radiograph shows normal periapical tissues.

A 47-year-old female patient visited the Endodontic Department of the University of Guadalajara, Mexico, for a checkup of the tooth #43 (right mandibular canine). She was referred by a general dentist, being asymptomatic, with caries located in the buccocervical region of the crown, which had reached the pulp chamber. The general dentist performed an initial cleaning of the caries two weeks prior, but given its depth and extent, the patient was referred for a specialized assessment. She had no history of systemic or allergy problems. In the clinical examination, a dental giroversion, with a maladjusted temporary restoration surrounded by recurrent cavities, was observed. Under magnification, it was confirmed that the caries was in clear communication with the pulp chamber. Response to the sensitivity test was intense and transitory. Radiographic examination revealed a sudden loss in the continuity of the canal (Figure 1(a)) and the presence of a groove in the outer part of the root, findings that suggest the presence of a mandibular canine with two independent, narrow, and curved canals. Radiographic images of the counterpart canine showed normal characteristics (Figure 1(b)). On the basis of the clinical and radiographic findings, a diagnosis of asymptomatic irreversible pulpitis and normal periapical tissues was established, and root canal treatment was planned.

After the patient provided signed informed consent, local anesthesia was administered and a dental dam was placed; the temporary restoration and decayed tissue were removed. As the dental giroversion impeded the lingual conventional opening, cameral access was created from the buccal side (Figure 1(c)). With the aid of magnification throughout the treatment, the pulp chamber roof and the lingual cervical ridge were eliminated to obtain access to the second canal. As the initial clinical and radiographic findings indicated the presence of a second root canal, the radicular pulp space was carefully screened with the DG-16 endodontic explorer (American Eagle, CA, USA), and the entrances of two root canals were found, one buccal and one lingual. On the basis of this information, the treatment protocol was focused on avoiding removal of excessive tooth structure or perforation of the roots. Under abundant irrigation with 1% sodium hypochlorite (NaOCl), the canals were explored with a size #15 K-file, and cervical flaring was carefully performed with #2 Gates Glidden bur. The working length was determined with a radiograph and corroborated with an electronic apex locator. Cleaning and shaping of the apical thirds were performed with rotatory NiTi files (S1, S2, and F1 Universal ProTaper, Dentsply, Tulsa, OK), followed by manual instrumentation with a size #35 Flexo-file in both root canals, irrigating with 1% NaOCl between the use of each instrument. Due to time constraints, intracanal dressing with calcium hydroxide was placed and the access was sealed with a temporary cement.

After 7 days, the intracanal medication was removed with 17% ethylenediaminetetraacetic acid (EDTA) irrigation and ultrasonic activation. Once both canals were dried, the lingual canal was first filled, to prevent visibility obstruction, followed by the buccal root canal. They were filled with a mix of gutta-percha and resin-based sealer (AH-Plus), using lateral cold compaction. The access opening was sealed with temporary cement, and a final radiograph was taken (Figure 1(d)). The 12-month follow-up showed the access cavity restored with a composite-based sealing material, with no pain or tenderness and normal periapical tissues upon clinical and radiographic examinations (Figure 1(e)).

## 3. Literature Review

### 3.1. Information Sources and Search Strategy

The electronic databases PubMed (https://www.ncbi.nlm.nih.gov/pubmed), Cochrane (http://www.cochrane.org), and Scientific Electronic Library Online (SciELO) (https://www.scielo.org) were searched to locate relevant articles (last access was on April 06, 2017). The following keyword combinations were used: “mandibular canine”, “two root canals”, “root canal anatomy”, and “anterior teeth”. Moreover, extensive manual search by two individuals (H. P. and M. D.) of four relevant scientific magazines (Journal of Endodontics, International Endodontic Journal, Oral Surgery Oral Medicine Oral Pathology Oral Radiology & Endodontics, and Dental Traumatology) was performed, including the reference section of each relevant article with the objective of finding additional works. Furthermore, endodontic textbooks and arbitrated and indexed magazines that did not appear in these electronic databases were inspected.

### 3.2. Selection Criteria

A total of 2324 articles were identified, 2266 in the electronic databases and 58 from the manual searches. After eliminating duplicate articles, the titles/abstracts of all manuscripts were reviewed to check if the articles fulfilled the following inclusion criteria, in sequential order:

1. Analyze the root canal anatomy of the mandibular canine.
2. Report the presence of mandibular canine with two roots and two root canals.

Subsequently, all the works were thoroughly and independently inspected by two expert endodontists (H. P. and M. D.). In cases where there was lack of concordance between the reviewers, a third reviewer made the final decision (B. R.).

### 3.3. Data Extraction

Of the 2324 articles initially identified, only 44 fulfilled both inclusion criteria. The 44 articles were classified according to the type of study as follows: clinical studies,* in vitro/ex vivo* studies, and case reports. From the clinical studies and the* in vitro/ex vivo *studies, the following information was obtained:

1. Study author(s)
2. Country were the study was conducted
3. Sample size (number of mandibular canines and their origin, if it was specified)
4. Method used for the study of the internal anatomy
5. Prevalence of two roots and two root canals in the mandibular canine
6. Root canal system configuration of the mandibular canine according to Vertucci's classification (Tables 1 and 2) [3].

For each case report, the following information was registered:

1. Study author(s)
2. Country were the study was conducted
3. Tooth
4. Patient age and sex
5. Root canal system configuration of the mandibular canine according to Vertucci's classification (Table 3) [3].

## 4. Discussion

This study aimed to present a case of a mandibular canine with two roots and two root canals and to review the available literature on this anatomical variation. In general, the mandibular canine is considered to have a high prevalence of Vertucci's type I configuration [2, 3, 14], which is in agreement with our result obtained from the literature review (85%) (Tables 1 and 2). However, the classic articles of Hess (1921, 1925) [50, 51] reported a noticeable low prevalence of single root canal in the mandibular canine (57.1%), which shows that anatomic variations in this tooth are a latent possibility. In fact, anthropologic findings have revealed that the mandibular canine with two roots was a common feature in Europeans from the 11th to the 19th century, in contrast to the null detection in the Asian population [52]. Such affirmation differs from the contemporary concepts, where it has been demonstrated that alterations in the number of roots and/or root canals tend to present higher prevalence in populations with Mongoloid features [53].

Without performing extensive imaging examinations, it is difficult to precisely determine by clinical means whether a mandibular canine presents only one root with two independent apical foramens or actually has two separate roots. Thus, the decision was made to group both alterations. With respect to this, only 18 articles (Tables 1 and 2) specified in detail the root canal system configuration of the mandibular canine, where the findings of two root canals and two independent apical foramina were the most common anatomical variations (5.7%). Two studies have focused on the morphological analysis specifically of two-rooted mandibular canines and evaluated diverse parameters of endodontic interest [54, 55].

Despite the fact that the mandibular canine is one of the least studied teeth, the first case report of finding a mandibular canine with two roots was published in 1886 (Table 3) [24]. We found 25 other reports on that entity of which 22 were published between the years 2000 and 2017. We found that the variation has a strong preference for the female sex (87.5%), mainly in patients with an average age of 38.1-years (range 17–60 years).

Early detection of a mandibular canine with two roots and two root canals favorably influences the success rate of endodontic treatment, as it allows the use of specific diagnostic tools and the setting of individualized strategies based on the anatomical particularity of the tooth. Therefore, it is crucial to carefully inspect the diagnostic radiographs; it is helpful to be aware of the importance of detection of a sudden loss in the continuity of the root canal lumen or a radiolucent groove in the lateral part of the root, which are findings that hint to the presence of more than one canal [56]. If a morphological alteration is suspected, acquiring angled radiographs (20°–25° or Clark technique) facilitates the detection of extra canals [57, 58]. If necessary, the use of limited field-of-view cone beam computed tomography will help to confirm the internal variations that are not clearly distinguishable with conventional methods [59]. Unfortunately, this technology also has some limitations, such as administration of a high radiation dose to the patient, possible artifact generation, high levels of scatter and noise, and variations in dose distribution, which should be considered when selecting the appropriate imaging modality in endodontic cases [60].

However, a mandibular canine with two roots can present bifurcation at different root levels, which implies a certain degree of difficulty according to the zone where it is located. When the division is located apically, there is a higher degree of difficulty during the localization and mechanical preparation of the root canals. In contrast, when the division is located more cervically, there are increased risks of perforating the bifurcation during the search for the additional canal. Therefore, performing an access opening in a straight line is crucial, as it improves visualization of the whole pulp chamber and aids with the determination of the accurate division point, preferably with the combined use of vision magnification and tactile examination with the DG-16 endodontic explorer. Moreover, it is useful to measure, through the root canal, the exact distance between the incisal reference point and the bifurcation zone, either with a periodontal probe or with a type K-file. Subsequently, by using ultrasound tips or long shaft burs, the pericervical dentin, which usually obstructs lingual canal access, should be carefully removed up to the distance that was previously determined, by performing small and progressively deeper movements. It is important to be careful with these types of movements, as they could excessively weaken the remaining root structure. The use of such strategies in combination with adequate operator skills could establish straight-line access to the root canals.

Versiani et al. (2011) [55] reported that in all two-rooted mandibular canines, the main apical foramen tends to be located eccentrically; therefore, the possibility of overinstrumentation is high. Thus, it is recommended to use an electronic apex locator to precisely locate the apical constriction. Furthermore, the timely detection of a mandibular canine with two roots and two root canals allows planning for a conservative mechanical instrumentation approach (apical size no larger than ISO #35), focusing on the preservation of the structure of the slim curved roots, which are susceptible to lateral perforation. Likewise, the abundant presence of accessory canals in this type of mandibular canine emphasizes the importance of adequate irrigation and obturation. According to Sharma et al. (1998) [54] and Versiani et al. (2011) [55], the prevalence of lateral communications and furcation canals is high, oscillating between 68.9% and 29%, respectively. Therefore, the use of final irrigation based on an apical negative pressure system, ultrasonic agitation, and 17% EDTA increases the removal of organic and inorganic tissue from the zones untouched by the instruments [61], which is complemented by the use of the continuous wave obturation technique for improving the sealing of the inaccessible anatomic zones [62].

## 5. Conclusion

Although the literature indicates that the occurrence of mandibular canines with two roots and two root canals is not common, these anatomical variations are associated with technical difficulties during endodontic treatment. Therefore, it is important to carefully inspect the preoperative radiograph for signs suggesting anatomical variations. Sudden loss in the continuity of the root canal lumen and a radiolucent groove in the lateral part of the root are findings that indicate the presence of more than one canal. Timely diagnosis and meticulous exploration of the internal anatomy of a mandibular canine with two roots and two root canals allow for the planning of an individualized treatment protocol, tailored to their peculiar morphology, focused on avoiding excessive weakening or perforation of the roots.

## Figures and Tables

**Table 1:** Prevalence of mandibular canines with two roots and two root canals reported in clinical studies (*n* = 5* articles*).

Author/year	Materials and methods	Country	Number of mandibular canines (*n*)	Root canal configuration^*∗*^	
				Type I (1) (%)	Prevalence of 2 apical foramina (%)
Aminsobhani *et al.* 2013 [4]	CBCT	Iran	608 (298 males and 310 females)	71.8%	19.8% (4.7% bi-rooted, 12.8% type IV [2], and 2.3% type V [1, 2])
Altunsoy *et al.* 2014 [5]	CBCT	Turkey	1604 (805 males and 799 females)	92.7%	3.9% (1.3% type IV [2] and 2.6% type V [1, 2])
Han *et al.* 2014 [6]	CBCT	China	1291 (645 right and 646 left)	93.7%	1.8% (1.3% bi-rooted and 0.5% type V [2])
da Silva *et al.* 2016 [7]	CBCT	Brazil	200	90.5%	4.5% (2.5% type IV [2] and 2% type V [1, 2])
Soleymani *et al.* 2017 [8]	CBCT	Iran	300 (172 females and 128 males)	89.7%	1% (all type V)

^*∗*^Vertucci's root canal configuration.

**Table 2:** Prevalence of mandibular canines with two roots and two root canals reported *in vitro/ex vivo* studies (*n* = 13* articles*).

Author/year	Materials and methods	Country	Number of mandibular canines (*n*)	Root canal configuration^*∗*^	
				Type I (1) (%)	Prevalence of 2 apical foramina's (%)
Pineda and Kuttler 1972 [9]	Periapical radiographs	Mexico	187	81.5%	5% (birooted)
Green 1973 [10]	Microscopic inspection of longitudinal sections	USA	100	87%	3% (type IV [2])
Kerekes and Tronstad 1977 [11]	Microscopic inspection of cross-sections	Norway	20	90%	10% (5% birooted and 5% type V [1-2])
Hession 1977 [12, 13]	Periapical radiographs and contrast dye	Australia	9	88.9%	11.1% (birooted)
Vertucci 1974 [14] and1984 [3]	Clearing and staining with China ink	USA	100	78%	6% (type IV [2])
Pécora et al. 1993 [15]	Clearing and staining with China ink	Brazil	830	92.2%	2.9% (1.7% birooted and 1.2% type IV [2])
Matzer 1993 [16]	Clearing and staining with China ink	Guatemala Republic	50	96%	4% (birooted)
Calişkan et al. 1995 [17]	Clearing and staining with China ink	Turkey	100	80.3%	1.9% (type V [2])
Sikri and Kumar 2003 [18]	Periapical radiographs and clearing	India	100	70%	12% (10% type IV [2] and 2% type V [1-2])
Sert and Bayirli 2004 [19], Sert et al. 2004 [20]	Clearing and staining with China ink	Turkey	200	76%	1.5% (type IV [2])
Oliveira and Lorio 2007 [21]	Periapical radiographs and clearing and staining with China ink	Brazil	1040	91.9%	1.1%
Rahimi et al. 2013 [22]	Clearing and staining with China ink	Iran	149	80.5%	12% (birooted)
Amardeep et al. 2014 [23]	CBCT	India	250	79.6%	2% (type V [1-2])

^*∗*^Vertucci's root canal configuration.

**Table 3:** Case reports of mandibular canines with two roots and two root canals (*n* = 26* articles*).

Author/year	Country	Tooth	Age (y)/sex	Root canal configuration^*∗*^
Vawter 1886 [24]	USA	#43	45 (female)	Birooted
Koskins 1926 [25]	USA	#33, #33, #33	35 (female), 35 (female), and 35 (female)	All type V (1-2)
Slowey 1974 [26]	USA	#33	Unspecified	Type IV (2)
Rahmatulla and Wyne 1993 [27]	Yemen	#43	28 (female)	Type V (1-2)
D'Arcangelo et al. 2001 [28]	Italy	#43, #43	48 (female) and 60 (female)	Both type IV (2)
Wang et al. 2009 [29]	China	#43, #43	46 (female) and 40 (female)	Type II (2-1) and IV (2)
Victorino et al. 2009 [30]	Brazil	Bilateral	40 (female)	Both type V (1-2)
Oporto et al. 2010 [31]	Chile	#33	37 (female)	Type V (1-2)
Maden et al. 2010 [32]	Turkey	#43	17 (female)	Type IV (2)
Andrei et al. 2010 [33]	Romania	#43	54 (female)	Type IV (2)
Gaikwad 2011 [34]	India	#33	20 (female)	Type V (1-2)
Fonseca et al. 2011 [35]	Brazil	#33	39 (male)	Type V (1-2)
A. Bhardwaj and A. Bhardwaj 2011 [36]	India	#43	18 (female)	Type V (1-2)
Andrei et al. 2011 [37]	Romania	#43	59 (female)	Type IV (2)
Saberi 2011 [38]	United Kingdom	#43	30 (male)	Type V (1-2)
Moogi et al. 2012 [39]	India	#43	32 (female)	Type V (1-2)
Batra et al. 2012 [40]	India	#33	35 (female)	Type V (1-2)
Ramírez-Sotelo et al. 2013 [41]	Brazil	#33	21 (female)	Finding of birooted mandibular canine in CBCT and panoramic radiograph
Mithunjith and Borthakur 2013 [42]	India	#43	42 (female)	Type IV (2)
Fuentes and Borie 2013 [43]	Chile	Bilateral	44 (female)	Finding of birooted mandibular canines in panoramic radiograph
Stojanac et al. 2014 [44]	Serbia	#33, #33, #33, #43	42 (male), 38 (female), 44 (female), and 46 (female)	Types V (1-2), IV (2), V (1-2), and IV (2)
Mukhaimer and Arandi 2014 [45]	Palestine	#43	23 (female)	Type V (1-2)
Ganesh et al. 2014 [46]	India	#33	46 (female)	Type IV (2)
Gandhi and Bhat 2014 [47]	India	#43	39 (male)	Type IV (2)
Fumei et al. 2014 [48]	Italy	#43	30 (female)	Type V (1-2)
Kulkarni et al. 2016 [49]	India	#43	54 (female)	Type IV (2)

^*∗*^Vertucci's root canal configuration.
